# Supplementary material for: Prevalence of previously diagnosed diabetes and glycemic control strategies in Mexican adults: ENSANUT-2016
Source: PLoS One. 2020 Apr 16;15(4):e0230752. doi: 10.1371/journal.pone.0230752 (PMC7162504; doi:10.1371/journal.pone.0230752)
Supplement: Attachment — Submitted filename: Respuesta a revisores - 240120.docx [file pone.0230752.s001.docx]

**RESPONSE TO REVIEWER'S COMMENTS**

**PONE-D-19-29166
Prevalence of previously diagnosed diabetes and glycemic control strategies in Mexican adults: ENSANUT-2016**

**Comments to the author.**
1. Is the manuscript technically sound, and do the data support the conclusions?
Reviewer #1: Partly

2. Has the statistical analysis been performed appropriately and rigorously?
Reviewer #1: I Don't Know

3. Have the authors made all data underlying the findings in their manuscript fully available?
Reviewer #1: Yes

 4. Is the manuscript presented in an intelligible fashion and written in standard English?
Reviewer #1: Yes

**Reviewer´s comment 1.** Abstract: There is a typo on line 26, pertaining to the year of the ENSANUT survey.

**Authors response**: We appreciate the observation. We have corrected the name of the survey and now "ENSANUT-2016" appears.

**Reviewer´s comment 2.** Unclear whether odds ratios are crude or adjusted for other factors.

**Authors’ response**: We clarify that in Table 2, in the last row it is described that OR are “adjusted for sociodemographic, anthropometric and clinical variables from the table”.

In the abstract, we now specify that the OR are adjusted: “The **adjusted** OR for having diabetes was higher in adults aged ≥60 years (OR = 1**1.0** in women and OR = **30.7** in men) than in adults aged 20-39 years (OR=1.0). The **adjusted** OR for having diabetes was higher in overweight men (OR=1.**7**) than in men with normal BMI (OR=1.0).”

**Reviewer´s comment 3.** On lines 34-36, it is unclear whether the percentages reported refer to all ENSANUT participants or only those diagnosed with diabetes. Also, if 44.9% measured their venous blood glucose, how is it possible that **69.5**% were estimated as not engaging in any control strategies? The two percentages would exceed 100%.

**Authors’ response**: We appreciate the observation. We have corrected the data and now the following appears: “A total of **30.5**% of the participants did not report any control strategies, 44.9% measured their venous blood glucose...,”.

**Reviewer´s comment 4.** The discussion should be based on reported results, not report new results. The authors mention activity and education, but those results are not reported in the results section.

**Authors’ response**: The authors have attended this comment. Now the risk factors described in the results section are the same as those included in the conclusions section (age and overweight).

**Reviewer´s comment 5.** The discussion also mentions that half of participants used control strategies but that percentage is not reported in the results section either.

**Authors’ response**: In response to this observation in results section we add: “A total of 30.5% of adults with diabetes did not report any control strategies, 44.9% measured their venous blood glucose, and 15.2% used the HbA1C as an indicator of glycemic control”

**Reviewer´s comment 6.** Introduction. It should be made clear whether the paper focuses on Diabetes Type 2 or any type of diabetes.

**Authors’ response**: Now we specify that the manuscript is focused on type 2 diabetes

**Reviewer´s comment 7.** Introduction. Why is the ENSANUT 2012 reported as the last national health survey? Shouldn’t it be ENSANUT 2016?.

**Authors’ response**: We appreciate the observation and to avoid confusion we have now written it as follows: "according to the national health survey 2012 ..."

**Reviewer´s comment 8.** Methods. Line 71: I don’t think the word “considering” is appropriate here. I think the authors probably mean that the survey “achieved” a 91.7% response rate. Please, double check.

**Authors’ response**: We appreciate the suggestion and now we include in the text "achieved”

**Reviewer´s comment 9.** Methods. Line 86: Did these questions inquire about lifetime diagnosis or within a certain time window?

**Authors’ response**: Now we specify in methods that the diagnosis of these pathologies refers to “throughout life”.

**Reviewer´s comment 10.** Methods. Lines 106-109: The description of the DD score categorization into recommended and non-recommended foods is confusing. These dietary measures need to be explained more clearly. Perhaps some examples would help.

**Authors’ response**: Now we specify: “... recommended (for example; fruits, vegetables, legumes, tubers, cereals with fiber, dairy, sugar-free drinks) and non-recommended foods (cereals with sugar, sweets and desserts, sugary drinks, alcoholic beverages, dairy drinks with sugar and processed meats).”

**Reviewer´s comment 11.** Methods. Lines 110-112: The authors also need to specify if questions about glycemic control strategies were asked of all participants or only those who reported a diabetes diagnosis. If only those with diabetes were administered these questions, the n sizes should be added to the first row of Table 3. If this information was obtained from everyone, it would make sense to stratify the results about glycemic control by previous diagnosis of diabetes, as they can expect to vary significantly by disease status.

**Authors’ response**: We have added the description that the questions about glycemic control “were self-reported only by participants previously diagnosed with type 2 diabetes”. On the other hand, Table 3 includes the “n” of each category.

**Reviewer´s comment 12.** Statistical Analyses. Please, unpack the variables used for adjustment, to increase replicability of the study. The list of variables adjusted for should also be reported as a footnote under Table 2.

**Authors’ response**: Now in Table 2 the variables that were used to adjust are shown as footnotes: “Adjusted OR for sociodemographic variable (sex, age, socioeconomic tertile, education level, area of residency and region); anthropometric (body mass index); prior medical diagnosis (high blood pressure, cerebrovascular disease, acute myocardial infarction or angina and kidney failure); and lifestyles (physical activity, screen time, dietary diversity, smoking and sleep quality).

**Reviewer´s comment 13.** Results/Tables. It appears, per the 95% CI, that women with primary or less education were also more likely to be diagnosed with diabetes than their male counterparts, but this result is not reported.

**Authors’ response**: Now we describe in the manuscript the following: " Women with primary or less education were also more likely to be diagnosed with diabetes (18.8%; CI95% 14.9-23.4) than their male counterparts (12.5%; CI95% 10.4-14.8)”.

**Reviewer´s comment 14.** Results/Tables. The result reported for gender differences among those with overweight or obesity (Lines 128-129) seems at odds with the overlapping 95% CIs shown on Table 1. Is that result after adjustment for other factors? If so, this should be stated.

**Authors’ response**: We appreciate the observation. Because 95% CIs overlap, we decided to exclude that description: “When identifying adults with overweight or obesity, the prevalence of diabetes was 30% higher in women than in men”.

**Reviewer´s comment 15.** Results/Tables. Table 1 should show the prevalence rates among those with and without the conditions listed under “Prior Medical Diagnosis”. That is, prevalence for those with and without a diagnosis of high blood pressure; with and without overweight or obesity; etc.

**Authors’ response**: Now the Table 1 shows the prevalence with or without the disease.

**Reviewer´s comment 16.** Results/Tables. The reporting of results shown on Table 2 (Lines 145-151) can be significantly improved. I recommend reporting the results separately for women and men and being more systematic listing all of the factors that were statistically associated with a diabetes diagnosis after adjustment for other confounders, instead of picking/choosing just a few.

**Authors’ response**: The authors appreciate the suggestion, however, we believe that the description included shows the most relevant findings. To provide more detail we have added the result of interactions: “In an adjusted model we tested interactions between each of the included variables and sex, observing significant interaction (p <0.055) only with age, socioeconomic tertile and education level."

**Reviewer´s comment 17.** Results/Tables. I would like to see the results for all (women and men combined) on Table 2, just like Tables 1 and 3. If it’s difficult to fit all of the information on one table, the authors could consider presenting only adjusted results and provide the unadjusted ORs as supplemental material?

**Authors’ response**: We attended this comment and now we include the combined results in Tables 1, 2 and 3.

**Reviewer´s comment 18.** Results/Tables. On Table 3, it is unclear what “no testing” means. As stated in one of my comments for the abstract, if 69.5% did not testing, how is it possible that 44.9% did venous blood sampling? This should be mutually exclusive categories, yet they add up to more than 100%.

**Authors’ response**: We appreciate the valuable comment. We review each value in the table and now we include the correct data: “... A total of 30.5% of adults with diabetes did not report any control strategies, 44.9% measured their venous blood glucose...”

**Reviewer´s comment 19.** Discussion. Please, comment on how the estimated prevalence compares to estimates based on earlier ENSANUT surveys, to give a sense of any potential trends.

**Authors’ response**: We have added the following information: “The prevalence of previous diagnosis of diabetes in Mexico increased from 7.0 to 9.2% between 2006 and 2012,[8] however, in the following six years (2016) the increase was only 0.2%.[11] This reflects that fewer people with diabetes are unaware of having this disease and the timely diagnosis has improved in recent years”.

**Reviewer´s comment 20.** Discussion. Lines 207-208: Please, double check and clarify why these percents exceed 100%.

**Authors’ response**: We appreciate the valuable comment. We review each value in the table and now we include the correct data. The percentage described before was 69.5%, but the correct was the complement (30.5%).

**Reviewer´s comment 21.** Discussion. Lines 219-220: Clarify what “sub-diagnosis” means here and revise this sentence to make its meaning clearer. Is this about hypertension or diabetes?

**Authors’ response**: To avoid confusion we have changed the description as follows: “...Even though 49.2% of Mexican adults had hypertension. [27] Of the adults analyzed in this study, only 6.1% verified their blood pressure; therefore, it is possible that there is a high percentage of adults with hypertension and are unaware of having this disease. This would increase the risk of developing associated complications if this situation is not reversed in the short term.

**Reviewer´s comment 22.** Discussion. Lines 224-225: This sentence is unclear. Is this still based on ENSANUT 2016? Also, the issue of social desirability bias should be addressed separately, as part of the limitations, as it can apply to all of the self-report based data used for this analysis.

**Authors’ response**: To avoid confusion:

1) We now specify that the result is based on ENSANUT 2016.
2) The reference to social desirability bias was moved to the limitations section. Now we describe this as follows: “We recognize that our results may be influenced by the possible measurement bias that represents the use of a self-report and by the social desirability bias in answering the questionnaires”.

**Reviewer´s comment 23.** Discussion. Line 231-233: The authors could cite other studies that have compared measured versus self-reported diabetes for Mexican adults, even if outside Mexico, as further evidence of likely undestimatio of true prevalence of diabetes. See Barcellos et al. Health Affairs 2012;31(12).

**Authors’ response**: We appreciate the suggestion of the reference and the comment.

We have now added the following paragraph to the discussion: "... in Mexican immigrants participating in the National Health and Nutrition Examination Survey when self-report is used as a diagnostic tool, only half of adults with diabetes (11.3%) are detected compared to using glucose measurement as a complementary method (22.6%).[33]

**Reviewer´s comment 24.** Discussion. A paragraph with implications and/or recommendations for future research and practice would strengthen the discussion, as would adding a little bit of context regarding any national strategies to improve diabetes prevention and control.

**Authors’ response**: To respond we have added the following paragraph in the discussion: “The high prevalence of diabetes found in our study should motivate in the short term estimate the total prevalence of diabetes including glucose measurement as a diagnostic method. We also believe it is necessary to evaluate the suitability of current programs for the diagnosis, prevention and control of diabetes such as PrevenIMSS and PrevenISSSTE to reduce its prevalence and improve glycemic control”.
