# Supplementary material for: Prevalence of previously diagnosed diabetes and glycemic control strategies in Mexican adults: ENSANUT-2016
Source: PLoS One. 2020 Apr 16;15(4):e0230752. doi: 10.1371/journal.pone.0230752 (PMC7162504; doi:10.1371/journal.pone.0230752)
Supplement: Attachment — Submitted filename: Response to Reviewers_ 260220.docx [file pone.0230752.s002.docx]

**RESPONSE TO REVIEWER'S COMMENTS**

**PONE-D-19-29166R1
Prevalence of previously diagnosed diabetes and glycemic control strategies in Mexican adults: ENSANUT-2016**

**Comments to the author.**

1. If the authors have adequately addressed your comments raised in a previous round of review and you feel that this manuscript is now acceptable for publication, you may indicate that here to bypass the “Comments to the Author” section, enter your conflict of interest statement in the “Confidential to Editor” section, and submit your "Accept" recommendation.
Reviewer #1: All comments have been addressed

2. Has the statistical analysis been performed appropriately and rigorously?
Reviewer #1: Yes

3. Have the authors made all data underlying the findings in their manuscript fully available?
Reviewer #1: Yes

 4. Is the manuscript presented in an intelligible fashion and written in standard English?
Reviewer #1: Yes

**Comments to the Author, 1.**

The description of the dietary measure is still unclear. I understand how the initial DD score was computed (number of food groups x number of days they were consumed) and the use of quartiles to create four groups from lowest to highest dietary diversity, but I am confused about the classification of food groups into desirable and not desirable. How was the classification of the food groups into these categories factored in when computing the DD score and quartiles?

**Authors response**: We appreciate the observation and we apologize because we had not updated this information.

We inform you that the classification of food groups (desirable and undesirable) was not considered in the final analysis. We only consider the score (quartiles) described by the reviewer (number of food groups x number of days they were consumed). Therefore, we have deleted the text associated with desirable and undesirable foods.

**Comments to the Author, 2.** I would like to see the findings regarding dietary diversity as a protective factor against a diabetes diagnoses reported in the results section and commented on the discussion. It seems to me this is an important result with implications for public health campaigns promoting dietary improvements. It is also interesting that the pattern of results for this factor is different for men and women, with a significant association for women, but not for men.

**Authors’ response**: To answer this comment we now include the following paragraphs in results and discussion:

Results : “When we compare the diversity of consumption of food groups or DD, we observe that in the total population and women with the highest quintile of DD (fourth quintile) the OR of having diabetes was lower (total population 0.5 CI 95% 0.3-0.7; women 0.4 CI 95% 0.2-0.7) than in the first quintile (OR = 1.0)”.

Discussión: “Dietary management is important to prevent diabetes [30] and dietary diversity is inversely associated with the risk of developing diabetes. [31] In our analysis we found that in women and total population having a greater DD was associated with a lower probability ratio of being diagnosed with diabetes. In men we do not find that DD is associated with a lower risk of diabetes possibly because in some subpopulations such as Hispanics [32] the results are still inconsistent and it is necessary to use a methodology that measures DD more accurately.These findings should motivate the generation of new studies that analyze this association longitudinally to confirm the direction and magnitude of causality. If this association is confirmed, it would be advisable to design communication programs to promote DD as another strategy to prevent diabetes”.

**Reviewer´s comment, 3.** On Table 1, the estimates for individuals without kidney failure are missing.

**Authors’ response**: We appreciate the observation. We have added the missing values on Table 1.

**Reviewer´s comment 4.** On line 195, the “six” years appears incorrect. It should be 10 (if the reference point is 2006) or 4 (if the reference point is 2012).

**Authors’ response**: The authors have attended this comment. Now the text describes that it was four years.

**Reviewer´s comment 5.** On line 227, there seems to be a typo. I think the authors mean “…with hypertension who are unaware”.

**Authors’ response**: We have modified the text and now the following is included: "... with hypertension who are unaware... “

**Reviewer´s comment 6.** The sentence on lines 259-261 needs to be revised to make sense.

**Authors’ response**: The authors have attended this comment. Now we describe the following: “One of the strengths of the study is that the results are representative of the Mexican adult population and are the most recent data on the prevalence of diabetes. This information will help the decision makers in health policies to know the magnitude of this disease, main associated risk factors and diabetes control practices.”
